# The Impact of Paulownia Leaves Extract on Performance, Blood Biochemical, Antioxidant, Immunological Indices, and Related Gene Expression of Broilers

**DOI:** 10.3389/fvets.2022.882390

**Published:** 2022-07-05

**Authors:** Shimaa A. Sakr, Huda A. EL-Emam, Mohammed A. E. Naiel, Noha M. Wahed, Hanan A. Zaher, Mohammed Sh. Abougabal, Youssef S. Alghamdi, Sarah Albogami, Mohamed Mohamed Soliman, Mustafa Shukry, Mona M. Elghareeb

**Affiliations:** ^1^Department of Husbandry and Development of Animal Wealth, Faculty of Veterinary Medicine, Mansoura University, Mansoura, Egypt; ^2^Department of Animal Production, Faculty of Agriculture, Zagazig University, Zagazig, Egypt; ^3^Food Hygiene and Control Department, Faculty of Veterinary Medicine, Mansoura University, Mansoura, Egypt; ^4^Department of Animal Production, Faculty of Agriculture, Al-Azhar University, Cairo, Egypt; ^5^Department of Biology, Turabah University College, Taif University, Taif, Saudi Arabia; ^6^Biotechnology Department, College of Science, Taif University, Taif, Saudi Arabia; ^7^Clinical Laboratory Sciences Department, Turabah University College, Taif University, Taif, Saudi Arabia; ^8^Physiology Department, Faculty of Veterinary Medicine, Kafrelsheikh University, Kafr El-Sheikh, Egypt; ^9^Physiology Department, Faculty of Veterinary Medicine, Mansoura University, Mansoura, Egypt

**Keywords:** paulownia extract, growth performance, immunity, broiler, antioxidant

## Abstract

The current research sought to assess the effects of paulownia leaves extract (PLE) on performance, blood hematological, antioxidant activity, and immunological response of broiler chicken. In total, two hundred 1-day-old male *Cobb*^500^ chicks were allocated randomly into four equal treatments with 5 replicates. The first treatment served as a control (CNT) and was fed the basal diet only, while the other treated treatments were fed on the basal diet supplemented with 0.1, 0.3, and 0.5 g/kg diet of PLE, respectively. The performance results showed significant increments (*P* < 0.05) in live body weight (LBW), weight gain (WG), and European production efficiency factors (EPEIs) (linearly; *p* < 0.001) in cooperated with increasing PLE levels in broiler diets. At the same time, feed conversion ratio (FCR) and livability percentages were numerically enhanced under the effects of PLE supplementation. Moreover, a notable increase (*P* < 0.05 or 0.01) in oxidative remarks activity (GSH, glutathione; SOD, super oxide-dismutase and CAT, catalase) and elevated levels of immunoglobulin (IgM, immunoglobulin M and IgG, immunoglobulin G) were noted (*P* < 0.05) for treatments fed with PLE in a dose-dependent manner. Also, a dramatic linear increase was observed in *mRNA* expression of *IGF-1, GHR, IL-1*β, and *IL-10* genes of broiler chickens. This study concluded that enriched broiler feeds with 0.5 g/kg PLE might be a beneficial strategy to promote broiler health and production.

## Introduction

For many decades, the poultry production industry has been under growing pressure to enhance productivity to offer high-quality meat products to feed the world's rising population while dealing with limited natural resources. Disease outbreaks may affect poultry growth and feed efficiency directly by reducing the digestive tract's capability of absorbing all nutrients and indirectly by limiting microbiota diversification and development (1). Therefore, employing antibiotic products as a feed treatment in poultry diets to alleviate infectious disorders or as growth promoters has resulted in the development of a large number of antibiotic-resistant bacterial strains that may threaten consumer health (2). Thus, because of consumer health concerns, certain antimicrobial growth promoters (AGPs) have been totally restricted internationally, particularly, in the European Union, since 2006 (3). Eventually, the ban of AGP in poultry production intensified the search for alternatives to improve the health and productive efficiency of broiler chickens (2, 4). Recently, some feed additives such as prebiotics, probiotics, essential oils, enzymes, and organic acids have been applied to replace AGP in poultry diets (5). The natural feed additives could enhance appetite and consumed feed, elevate higher nutrient digestibility, increase digestive enzyme activity and secretion, maintain the intestinal microvilli, antimicrobial activity, antifungal activity, antiviral activity, antioxidant effects, and immune stimulant action (6, 7).

Specifically, supplemented poultry feed with several phytogenic molecules extracted from plants improved performance and boosted general health status (1), for instance, *Prunus armeniaca* (8) and *Hippophae rhamnoides* (9). In addition, enriched animal and poultry diets with phytogenics promoted performance, feed efficiency, appetite, carcass meat quality, and health status (10, 11).

Paulownia (*P. tomentosa*) is one of the most exploited medicinal plants that grow natively in China, Japan and the Far East Asian countries (12, 13). Besides, it has been used in traditional Chinese medicine to treat or prevent a wide range of infectious diseases (14). He et al. (15) indicated that each of paulownia parts (seeds, roots, wood, fruits, flowers, bark, and leaves) had been exhibited to have one or more bioactive molecules, for instance, matteucinol and ursolic acid (leaves), dsesamin and paulownia (wood), catalpinoside, and syringin (bark). Furthermore, the high-flavonoid concentration of paulownia elongate leaf extracts, both dried and fresh, suggests that this plant has the potential for novel therapeutic applications against a broad range of oxidative disorders (12). Therefore, paulownia leaves, fruits, and flowers (a wood by-product) are the most important plant parts employed in the folk herbal medicine (16, 17). According to a Chinese medical book edited by Li Shizhen in 1578, paulownia bark might be applied to treat hemorrhoids and worms infections, while the flowers could be used to reduce swelling and promote hair development (16).

Also, paulownia leaves might be employed as an alternate feed component for a variety of animals due to their diverse biochemical features (12). The main bioactive compounds found in paulownia (such as phenolic compounds, glycosides, flavonoids, lignans, saponins, syringin, and triterpenoids) exhibited several health benefits to consumers and animals (16, 18). In addition, paulownia and its extract showed several therapeutic properties as antibacterial, anti-inflammatory, thirst-quenching, diuretic, antihypertensive, hemostatic, and insecticidal agents (13, 19, 20). Furthermore, it could be considered a growth promoter and immunostimulant agent at various concentrations in animal diets (12, 21). Also, several recent investigations have shown the antibacterial and antioxidant characteristics of PLE-derived compounds loaded with chitosan or calcium alginate, and also their specific applicability in meat preservation (22, 23).

Paulownia leaves as a potential feed ingredient for domestic animals is a relatively new approach and research field (4). In the same context, a wood by-product of the paulownia tree has been utilized as an alternative feed ingredient for different animals due to its good nutritional value present in leaves and other parts (11). In addition, their varied biochemical properties are recognized for medical applications (24, 25). Still, too little research has focused on the bioactive chemicals derived from paulownia leaves and their positive impact on animal health. We hypothesized that owing to their effective antioxidant activity, PLE can effectively reduce the oxidative stress, boost immunity, and promote growth of broiler chicks. Thus, it is essential to determine bioactive components in PLE and evaluate the feasibility of supplemented broiler chicken diets with PLE as a natural growth promoter. Therefore, this study was designed to identify common bioactive components extracted from paulownia leaves and analyze their biological effects as feed additives on growth performance, blood hematology, antioxidant activity and immune response of broiler chickens.

## Materials and Methods

### Animal Ethical Approval Statement

All *in-vivo* trials were followed the practical guidelines of the Local Experimental Animal Care Committee and approved by the Ethics Committee of Animal Use in Research Committee of Mansoura University (Code No: R/87).

### Prepartion and Characterization of Paulownia Leaf Extracts (PLEs)

#### Plant Source and Collection

The leaf parts of paulownia (*Paulownia tomentosa*) was picked and gathered directly from trees (6 months old) from a wood tree farm located in Bani–Salama village, Wadi El-Natroun district, Beheira Governorate, Egypt. Fresh paulownia leaves were transported to laboratory for further processing. The collected leaves were washed, cleaned, air-dried, ground, then kept in an airtight container to avoid the effect of humidity and then stored at room temperature until further extraction and isolation.

#### Samples Preparation and Extraction

The dried leaves were made as a fine powder for extraction uses. Before extraction, about 20 g of the paulownia leaf powder was defatted by soaking in 120 ml methanol for 24 h in a rotatory shaker. The defatted dry plant material was then extracted in an ultrasonic bath (Sonicator-10 L, India MART, Inter MESH Ltd.) for 20 min with 300 ml of aqueous ethanol (50%, v/v). After extraction, the solution was filtered and concentrated through Whatman No. 1 filter paper to separate the extract of plant leaves. The extract was stored in the refrigerator for further phytochemical analysis followed Nety et al. (26) procedure.

#### Gas Chromatography–Mass Spectroscopy Analysis

In total, 1 mg of the dried leaf extract residue was dissolved in 1 ml of methanol and the extract was analyzed with Gas Chromatography–Mass Spectroscopy analysis (GC–MS). About 1 μl of the methanol extract was injected into the GC–MS using a micro-syringe. GC–MS analysis was carried out on the GC–MS-5975C Agilent system comprising an autosampler and gas chromatograph interfaced to a mass spectrometer utilizing the following condition. The sample was inoculated into the injected port of the gas chromatography (GC) device. The GC apparatus vaporizes the injected sample and after that, isolates and investigates the various components. Each component produces a certain spectral peak which will be recorded on a paper chart electronically. The time elapsed between elution and injection is called the “retention time.”

Before testing the extract using gas chromatography and mass spectroscopy, the oven temperature, the flow rate of the gas used and the electron gun were programmed initially. The identity of the components in the extracts was accomplished by the comparison of their retention times (RTs) and mass spectra fragmentation patterns with the help of a commercial standard mass spectral database. Only the components with a similarity index of 90% and above according to databases were considered (27, 28). The total phenolic content and total anthocyanin content were determined, followed Chattuwatthana and Okello (29) and Zakaria et al. (30) procedures, respectively.

#### Experimental Design, Management, and Feeding Regime

The present study was conducted at the Poultry Research Farm belonging to the Faculty of Veterinary Medicine, Mansoura University, Egypt. A total number of 200 1-day-old male broiler chicks *Cobb*^500^ were purchased from a local commercial hatchery. At an average 53.8 ± 0.54 g initial weight, 1-day-old chicks were randomly distributed into four equal experimental treatments (*n* = 50) with five replicates (10 × 5). The first treatment was fed the basal diet only without any PLE supplementation (served as a control), while the second, third, and fourth treatments were fed on a basal diet supplemented with 0.1, 0.3, and 0.5 mg g/kg diet of PLE, respectively. The concentrations examined were selected based on the results of Yang et al. (17).

All the birds were housed and reared under identical environmental, managerial, and hygienic conditions. A routine vaccination schedule against the most common viral diseases in Egypt was administered and necessary medication when needed based on diagnoses and symptoms shown by the birds (Table 1). The birds had free access to feed and water for *ad-libtum* consumption during the experimental period. The ambient temperature was gradually decreased from 32°C at placement to 21°C at 42 d and the light schedule decreased from continuous light (24 h) for the first 3 d to 16L:8D thereafter. For the first 3 weeks, chicks were fed on a starter ration (2,900 Kcal. ME/kg, 23% C.P.) and followed by a finisher ration (3,050 Kcal. ME/kg, 22% C.P.) for the remaining period. The basal diets were formulated to meet the requirements of the broiler strain (31) (Table 2).

**Table 1:** Vaccination program of the broiler.

**Age (day)**	**Disease**	**Vaccine**
1	Newcastle + infectious bronchitis	Vitabron-L
12	Infectious bursal disease (Gum-boro)	Cevac-IBD L
21	Newcastle + infectious bronchitis	Cevac-BI L

**Table 2:** Diet formulation and chemical analysis.

**Ingredients**	**Starter diet (1–21d)**	**Finisher diet (22–42d)**
Maize	52.3	54.5
Corn gluten meal 30%	2.5	0
Corn gluten meal 60%	2.5	1.6
Canola meal	15	14
Poultry by product meal^a^	4	6
Soybean meal (Hi-Pro)	19	17.8
Poultry oil^b^	2	3.8
Limestone	1	0.9
Salt	0.1	0.1
Di-calcium phosphate	0.4	0.25
Sodium Bi carbonate	0.18	0.2
Lysine sulfate 70%	0.43	0.32
DL-methionine 99%	0.19	0.19
L-threonine	0.08	0.02
Premix^c^	0.32	0.32
Total	100	100
**Nutrients**, %		
ME^d^, Kcal/Kg	2,900	3,050
Crude protein	23	22
Crude fat	4.3	6.5
Crude fiber	2.7	2.6
Ash	6.2	5.7
Dig.^e^ lysine	1.3	1.19
Dig. argenine	1.4	1.29
Dig. metionine	0.63	0.62
Dig. cystine	0.28	0.27
Dig. trptophan	0.24	0.23
Dig. leucein	1.63	1.56
Dig. iso leucein	0.89	0.84
Dig. threronine	0.85	0.78
Dig. valine	0.94	0.89
Starch	33.4	34.2
Calcium	0.92	0.79
Available P	0.46	0.40
Sodium	0.20	0.20
Chloride	0.20	0.20

*^a^Poultry by-product meal, pet food grade, Griffin Industries Inc., Bastrop, Texas, 66.3% crude protein (CP), ^b^Poultry oil, Berg and Schmidt India, Hamburg, Germany, 99% crude fat, 8,700 Kcal metabolic energy, ^c^each 1 kg of mineral premix and vitamin contain; choline chloride 70%, betain HCL 98%, diclazuril 1%, kemzyme, vitamin A 20,000,000 I.U, vit-D3 6,000,000 I.U. vit-E 60,000 mg, vit-K3 4,000 mg, vit-B1 4,000 mg, vit-B2 12,000 mg, vit-B6 8,000 mg, vit-B12 20,000 mcg, nicotinamide 80,000 mg, biotine 200,000 mcg, folic acid 2,000 mg, Ca d-pantothenate 20,000 mg, manganese 150,000 mg, zinc 120,000 mg, iron 96,000 mg, copper 20,000 mg, iodine 2,000 mg, selenium 400 mg, ^d^ME, metabolizable energy; ^e^Dig. digestible*.

#### Growth Performance and Blood Biochemical Traits

During the experimental period, all the birds were subjected to the same strategy of data collection. Chicks were independently weighed at a weekly interval until the 5th week of age then the mean body weight of the pen was cumulatively calculated as an experimental unit. Feed consumption was recorded weekly until the marketing age on a replicate basis and dead birds (if any) were recorded. Consequently, body weight gain (BWG), total feed intake (TFI), feed conversion ratio (FCR), and mortality percentage were estimated from these data during the experimental period. The performance index for broilers (European Production Efficiency Factors; EPEF) was used as ascribed by Aviagen (32). EPEF were calculated according to the following formula:


$$\begin{array}{l} \text{European Production Efficiency Factors }\left(\text{EPEF}\right):EPEF\\ =\frac{\text{Livability }\%*\text{FBW }\left(\text{kg}\right)}{\text{Period }\left(\text{days}\right)\;*\mathrm{FCR}}*\;100 \end{array}$$

#### Blood Biochemical Parameters, Oxidative Remarks, and Immune Activity

At the end of the experiment (35th day), blood samples (2 birds/replicate) were collected from wing veins into clean tubes without coagulating. The samples were coagulated and centrifuged at 3,000 rpm for 15 min and the separated sera were collected in Eppendorf, frozen and stored at −20°C until further analysis. The following serum biochemical parameters were determined: total protein (g/dl), albumin (g/dl), globulin (g/dl), total cholesterol (mg/dl), triglyceride (mg/dl), high-density lipoprotein (HDL), low-density lipoprotein (LDL) (mg/dl), reduced glutathione (GSH), super oxide dismutase (SOD), lipid peroxidation malondialdehyde (MDA), and catalase (CAT) and immunoglobulins, namely, IgG and IgM were estimated in the blood by using commercial diagnostic kits provided by the Bio Diagnostic Co. (29 El-Tahrir St. Dokki, Giza, Egypt) and a spectrophotometer (Shimadzu, Japan).

#### RNA Extraction and cDNA Synthesis

RNA was extracted using 30–50 mg of liver and spleen tissues from experimental chickens using the QIAamp RNeasy Mini kit (Qiagen, Germany, GmbH) following the manufacturer's protocol. The integrity and concentration of the obtained RNA were determined with spectrophotometric NanoDrop^®^ ND-1000. The synthesis of the first strand of cDNA from the obtained RNA was achieved through the use of QuantiTect Reverse Transcription kit (Qiagen, Heidelberg, Germany) and the manufacturing procedures were applied.

#### Quantitative Real-Time PCR

Quantitative real-time PCR was determined using Rotor-Gene Q cycler (Qiagen, Heidelberg, Germany) by SYBR Green QuantiTect PCR kits (Qiagen, Germany). Relative expression of mRNA level was carried out for each gene. The sequence of the used primers is illustrated in Table 3. Two possible housekeeping genes (GAPDH and YWHAZ) were selected as they are commonly applied as based controls in target tissue expression investigations. The reaction mixture consisted of 12.5 μl of 2x SYBR Green PCR Mastermix, 0.25 μl of RevertAid Reverse Transcriptase (200 U/μl) (Thermo Fisher), 0.5 μl of each primer of 20 pmol concentration, 8.25 μl of water, and 3 μl of RNA template. The reaction was performed in a Stratagene MX3005P real-time PCR machine. The thermal cycling conditions were: initial denaturation at 95°C for 15 min for a number of 40 cycles followed by initial heat activation at 94°C for 15 s; primers annealing at 59°C for 1 min for *IL-10* gene, 60°C for 1 min for *IL-1*β and *GHR*, 65°C for 1 min for *IGF-1*; finally, elongation at 72°C for 30 s. The relative fold changes in the mRNA expression of the studied genes were calculated as recorded by Yuan et al. (33) through the comparative ^2−ΔΔCt^ method (Ct: cycle threshold).

**Table 3:** The sequence of tested primers applied in real-time PCR analysis.

**Gene**	**Forward 5′ > 3′**	**Reverse 5′ > 3′**	**Acc. No**.	**Ampl. size (bp)**
*IL-1β*	CCAGAAAGTGAGGCTCAACA	GTAGCCCTTGATGCCCAGT	KJ891452	591
*IL10*	CTGCACTTCTCTGAGCTGCT	CTTCCTCCTCCTCATCAGCA	NM_001004414	528
*GHR*	CAGCTGCTGTTGACCTTGG	CCAGTGCCAAGGTCAACAG	AH002706	4829
*IGF-1*	TACCACCAACTCAGAGCAGG	GGTTTTCTTCTGCCTTGGGG	NM_001085376	8975
*GAPDH*	GACGTGCAGCAGGAACACTA	TCTCCATGGTGGTGAAGACA	AJ312193	602
YWHAZ	TTGCTGCTGGAGATGACAAG	CTTCTTGATACGCCTGTTG	GCA_016699485.1	104

*IL-1β, interleukin 1 Beta; IL10, interleukin 10; GHR, growth hormone receptors: IGF-1, insulin-like growth factor 1; GAPDH, glyceraldehyde-3-phosphate dehydrogenase; YWHAZ, tyrosine 3-monooxygenase/tryptophan 5-monooxygenase activation protein, zeta polypeptide*.

#### Statistical Analysis

The data obtained were subjected to the one-way ANOVA using IBM SPSS Ver. 24. The GLM statistical model was as follows: X_ijkl_ = μ + A_j_ + *ei*.

Where, X_ij_ = an observational data, μ = overall mean, A_j_ = effect of PLE supplementation level, *e*_*i*_ = random error. The main effect of the PLE supplementation was the experimental unit. Tukey's multiple range test was used to compare means when a significant difference (*p* < 0.05) was detected. For broiler performance, five replicates pen per treatment (10 broiler chicks per replicate pen) served as the experimental unit followed Hernández-Ramírez et al. (34).

## Results

### The GC–MS Analysis of Paulownia Leaf Extracts

The results of the GC–MS analysis of main bioactive compounds found in paulownia leaf extract with their retention time (RT), and area percentage are presented in Table 4. Chromatograms GC–MS result indicates that the tested extract of paulownia leaf has a variety of bioactive compounds. The presence of 5 main peaks determined was as follows: the first main bioactive compound was thymol which was identified in the shortest RT (7.86) with the highest peak area (82.37%) and the last compound Octasiloxane was identified in much longest RT (38.59) and low percentage peak area (2.78) was observed. Among those 2 bioactive compounds, too many phytocompounds having different biological activities were confirmed such as α-Tocospiro (RT:26.59; 3.18%), Phytol (RT:19.04; 9.28), and Pentadecanoic acid observed at (RT:16.82; 2.785).

**Table 4:** The gas chromatography/mass spectrometry analysis of the main bioactive compounds found in paulownia leaf extracts (PLEs).

**Bioactive compound**	**Retention time**	**Peak area %**
Thymol	7.86	82.37
Flavonoids	10.77	1.65
Phytol	19.04	9.28
Pentadecanoic acid	16.82	2.39
Octasiloxane	38.59	2.78
Alkaloids	26.41	1.10
Saponins	29.96	1.43
Terpenoids	20.27	0.33
Phenolics	38.99	1.27
à-Tocospiro	26.59	3.18
Aldehyde	20.88	0.25
TPC as mg/g dried PLE	36.5 ± 0.81	
TAC as mg/g dried PLE	3.74± 1.12	

*TPC, total phenolic content; TAC, total anthocyanin content; PLE, Paulownia leaf extract*.

The phytochemical characterization of the PLE of phytochemicals molecules indicated the presence of saponins and phenolics, namely, flavonoids, with total phenolic content (TPC) and total anthocyanin content (TAC) values of 36.5 ± 0.81 and 3.74 ± 1.12 mg/g dried PLE, respectively (Table 4).

### Growth Performance

The PLE supplementation on the growth performance of broiler chicks during the experimental period is presented in Table 5. Clearly, results showed that there were significant differences (*P* < 0.05) in final body weight (FBW) and body weight gain (BWG) due to PLE supplementation. Furthermore, increasing the inclusion level of PLE from 0.1 up to 0.5 g/kg within the broiler diet increased the positive effect on LBW and BWG (linearly; *p* < 0.002). On the contrary, TFI, FCR, and mortality rates showed no significant differences (*P* > 0.05) under the effects of PLE dietary supplementation. Whereas, values of FCR were numerically improved in PLE-treated treatments (PLE_0.1_, PLE_0.3_, and PLE_0.5)_ in comparison with the non-treated treatment (CNT). Besides, mortality percentage reduced from 2.96% in the CNT treatment to 1.48, 0.00, and 0.00 for PLE-treated treatments (PLE_0.1_, PLE_0.3_, and PLE_0.5_), respectively. Herein, the EPEI level was significantly (linearly; *p* < 0.001) increased gradually by about 10.96, 12.24 and 17.19% for PLE supplemented treatments (PLE_0.3_ and PLE_0.5_), respectively, in comparison with the control treatment.

**Table 5:** Productive performance parameters of broilers as affected by PLE supplemented diets at different levels.

**Parameters**	**PLE (g kg-1)**				**SEM**	***P*** **-values**		
	**CNT**	**PLE_**0.1**_**	**PLE_**0.3**_**	**PLE_**0.5**_**		**Combined**	**Linear**	**Quadratic**
IBW (g)	53.8	53.8	53.8	53.6	0.54	0.552	0.279	0.412
FBW (g)	1890.1^b^	1999.7^a^	2009.1^a^	2047.6^a^	2.35	0.008	0.002	0.174
WG (g)	1836.3^b^	1945.9^a^	1955.3^a^	1994.0^a^	3.27	0.008	0.002	0.175
TFI (g)	2886.9	2963.2	3006.2	2993.6	2.96	0.447	0.171	0.434
FCR (g/g)	1.57	1.52	1.54	1.50	0.17	0.568	0.247	0.864
Mortality %	2.96	1.48	0.00	0.00	0.51	0.099	0.023	0.397
EPEI	333.1^c^	369.7^b^	373.9^ab^	390.4^a^	6.70	<0.001	<0.001	0.097

*PLE, paulownia leaf extract; CNT, control treatment fed un-supplemented diet; IBW, initial body weight; FBW, final body weight; WG, weight gain; TFI, total feed intake; FCR, feed conversion ratio. ^a, b, c^, within the same raw indicates to significance differences (p < 0.05) between means*.

### Blood Biochemical Parameters

The effects of PLE supplementation on blood biochemical parameters of broiler chicks are presented in Table 6. Generally, results showed no significant differences (*P* > 0.05) in blood biochemical parameters (total protein, albumin, globulin, total cholesterol, HDL, and LDL) in all the experimental treatments. While, lipid triglycerides values showed a significant (*P* < 0.05) reduction under the effects of PLE dietary supplementation. Blood triglyceride values reduced from 208.3 in the CNT treatment to 194.5 and 148.8 for PLE_0.1_ and PLE_0.3_, respectively.

**Table 6:** Blood biochemical measurements of broilers as affected by PLE supplemented diets at different levels.

**Parameters**	**PLE (g kg-1)**				**SEM**	***P*** **-values**		
	**CNT**	**PLE_**0.1**_**	**PLE_**0.3**_**	**PLE_**0.5**_**		**Combined**	**Linear**	**Quadratic**
Total protein (g/dl)	2.68	3.03	3.14	2.84	0.74	0.262	0.446	0.072
Albumin (g/dl)	1.54	1.78	1.83	1.66	1.01	0.174	0.347	0.046
Globulin (g/dl)	1.15	1.25	1.30	1.18	0.89	0.421	0.601	0.130
Triglyceride (mg/dl)	208.3^a^	219.4^a^	194.5^a^	148.8^b^	1.08	0.001	0.792	0.001
Total cholesterol (mg/dl)	101.1	109.9	99.4	96.0	2.49	0.245	0.247	0.219
HDL (mg/dl)	37.57	40.45	39.90	30.37	1.51	0.052	0.075	0.029
LDL (mg/dl)	23.37	31.18	30.03	21.97	1.76	0.514	0.720	0.028

*PLE, paulownia leaf extract; CNT, control treatment fed un-supplemented diet; HDL, high-density lipoprotein; LDL, low-density lipoprotein. ^a, b^, within the same raw indicates to significance differences (p < 0.05) between means*.

### Oxidative Remarks and Immune Status

Oxidative remarks parameters such as GSH, SOD, MDA, and CAT are indicators for assessing the oxidative stress status of birds. Results in Table 7 illustrated the antioxidant parameters of broiler chicks at 35 days of age as affected by different levels of PLE supplementation. Data demonstrated that PLE supplementation significantly (*P* < 0.01) affects antioxidant parameters (GSH, SOD, and CAT) of different broiler treatments. On the other hand, lipid peroxidation activity (MDA) was not significantly (*P* > 0.05) affected by the different levels of PLE (PLE_0.1_, PLE_0.3_, and PLE_0.5_) in comparison with the non-treated treatment (CNT). Whereas, the MDA values showed slightly decreased in all the PLE-supplemented treatments than in the control treatment.

**Table 7:** Oxidative remarks and immune status of broilers as affected by PLE supplemented diets at different levels.

**Parameters**	**PLE (g kg-1)**				**SEM**	***P*** **-values**		
	**CNT**	**PLE_**0.1**_**	**PLE_**0.3**_**	**PLE_**0.5**_**		**Combined**	**Linear**	**Quadratic**
**Oxidative remarks**								
GSH (mmol/ml)	2.51^b^	2.61^b^	3.70^a^	4.17^a^	0.26	<0.001	<0.001	0.367
SOD (nmol/ml)	361.8^a^	292.2^b^	368.6^a^	274.1^b^	1.63	0.001	0.020	0.450
MDA(mmol/ml)	6.48	4.59	5.37	5.91	0.15	0.064	0.671	0.020
CAT (mmol/ml)	4.21^bc^	5.18^ab^	3.44^c^	5.46^a^	0.31	<0.001	0.124	0.076
**Immune activity**								
IgM (mg/dl)	22.6^b^	24.2^ab^	26.0^a^	24.2^ab^	0.91	0.48	0.083	0.049
IgG (mg/dl)	1.45	1.74	1.27	1.82	0.19	0.067	0.369	0.403

*PLE, paulownia leaf extract; CNT, control treatment fed un-supplemented diet; GSH, glutathione; SOD, super oxide-dismutase; MDA, malonaldehyde; CAT, catalase; IgM, immunoglobulin M; IgG, immunoglobulin G. ^a, b, c^, within the same raw indicates to significance differences (p < 0.05) between means*.

Glutathione (GSH) concentration was increased significantly (*P* < 0.05) and gradually (linearly; *p* < 0.001) from 2.51 in the CNT treatment to 2.61, 3.70, and 4.17 for PLE supplemented treatments (PLE_0.1_, PLE_0.3_, and PLE_0.5_), respectively. However, a similar pattern was not seen for SOD and CAT, where values for the SOD parameter tended to be higher in the PLE_0.3_ supplemented treatment than in the other treated treatments (PLE_0.1_ and PLE0.5). An opposite trend was found in CAT values, where it was tended to be higher in PLE_0.1_ and PLE_0.5_ treatments than in other treated treatments (PLE_0.3_) and non-treated treatments (CNTs).

In addition, in regards to immune activity parameters (IgM and IgG), results showed that dietary supplementation of PLE had a significant (*P* < 0.05) positive effect on IgM. The immune activity of IgM increased from 22.6 in the CNT treatment to 24.2, 26.0, and 24.2 for PLE_0.1_, PLE_0.3_, and PLE_0.5_, respectively. Furthermore, IgG values showed numerically improved for the broiler treatments that received PLE_0.1_ and PLE_0.5_ (1.74 and 1.82, respectively) compared with the control treatment fed un-supplemented diets (1.45).

### Gene Expression

**Figure 1:**
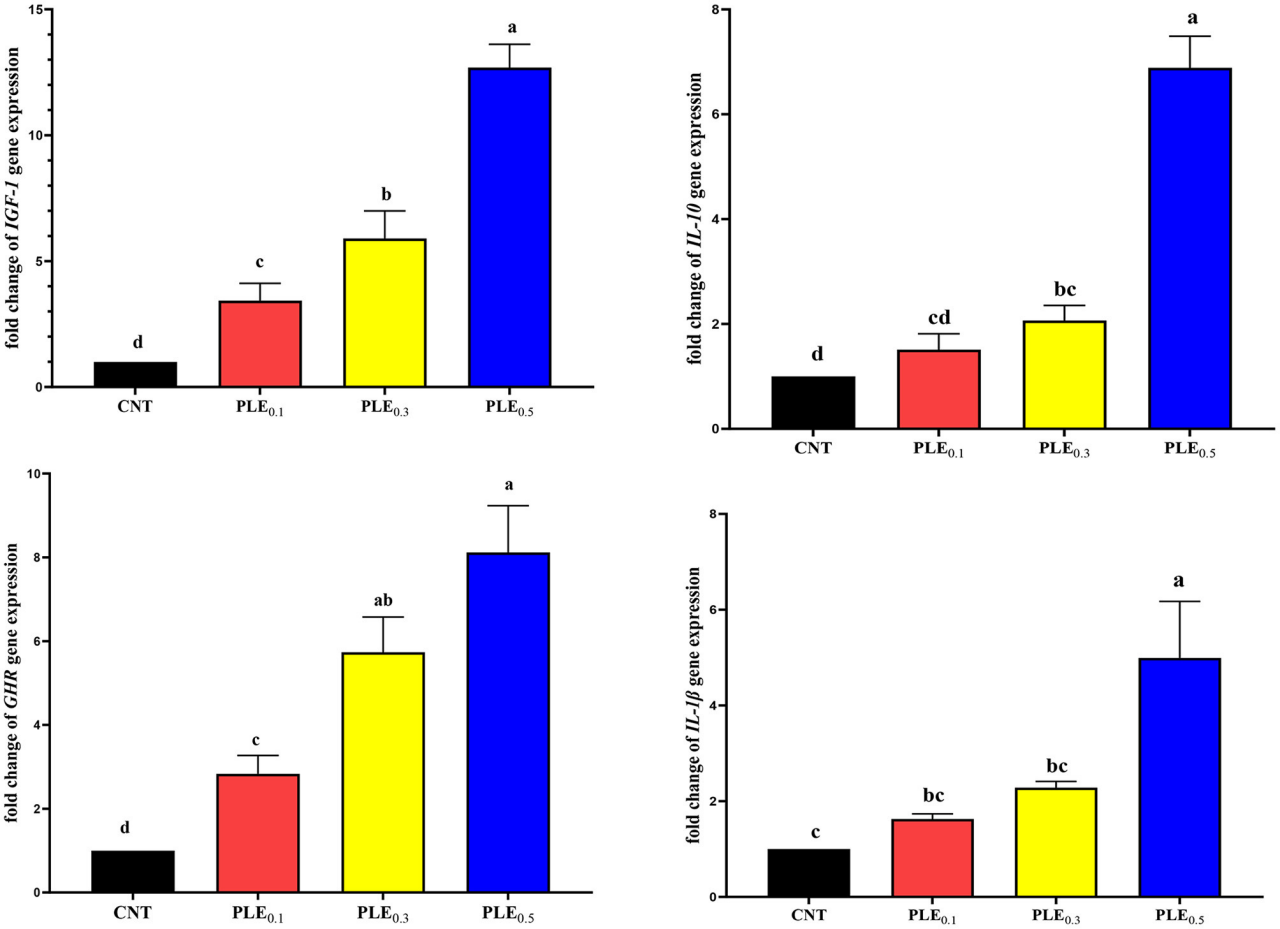
The effect of PLE dietary supplementation on the mRNA expression of the broiler chicken hepatic (*IGF-1* and *GHR*) and splenic (*IL-1*β and *IL-10*) genes (mean ± MSE). Columns with different superscript letters are significantly different (*P* < 0.05).

The effects of dietary supplementation of graded levels of PLE on mRNA expression of hepatic (*IGF-1* and *GHR*) and splenic (*IL-1*β and *IL-10*) of the broiler chickens are presented in Figure 1. The results showed a significant (*P* < 0.01) interaction between supplemented graded levels of PLE and the mRNA expression of all the assessed genes. A dramatic linear increase (*P* < 0.01) was observed in mRNA levels in hepatic *IGF-I* and *GHR* of birds fed with PLE in a dose-dependent manner. Compared with the un-supplemented treatment (CONT), the relative transcript levels of mRNA expression (*IGF-1* and *GHR*) in the liver were duplicated about 1, 2, and 3 times in treated broiler treatments supplemented with PLE_0.1_, PLE_0.3_, and PLE_0.5_, respectively. In the same manner, regarding splenic genes expression level, a significant upregulation in mRNA levels of splenic *IL-1*β and *IL-10* was observed with increasing the dietary level of PLE supplementation compared with the control treatment. Briefly, mRNA expression of all the evaluated genes was more prominent in broiler treatments with increasing PLE supplementation levels.

## Discussion

Paulownia is one of the traditional Chinese medical (13), which contains a variety of active ingredients such as flavonoids, phenolic acids, saponins, phenylethanoid glycosides, lignans, and triterpenoids in its leaf, fruit, and flower (12, 19, 35), with multiple pharmacological and economical values (18). Our GC mass analysis demonstrated that the methanolic extract of *P. tomentosa* included large amounts of thymol, phytol, flavonoids, and other bioactive compounds, which indicates the great efficacy of methanol as an organic solvent in extracting the primary bioactive molecules found within leaves. In addition, methanol is highly effective in extracting essential oils from plant material, particularly, the leaves (16, 19). Thus, this research studied the incorporation of *P. tomentosa* leaf extract into broiler diets and has shown that it is a viable strategy for improving performance and general health status.

Our feeding trial results showed that increasing supplemental levels of PLE resulted in noticeable linear increases in both final body weight and weight gain. The obtained results were found to be inconsistent with Agah et al. (36) findings that enriched broiler diets with olive leaf extract significantly promoted the growth and efficiency of consumed diets. In addition, Acamovic and Cross (37) demonstrated that supplementing broiler chicks' diet with a 1,000 mg/kg thyme essential oil significantly increased body weight growth as well as remarkedly reduced total feed intake. Thyme essential oils isolated from paulownia leaves showed higher antibacterial activities, which are particularly effective in promoting digestion through maintaining intestinal microbial balance (38) and stimulating the release of internal digestive enzymes (39), consequently enhancing overall growth performance in chicken.

In the same context, aromatic herbs and their extracts are well-known to enhance the flavor and palatability of feed, hence increasing growth and feed efficiency (40). In particular, thyme essential oil has a significant influence on feed conversion ratio because it could improve beneficial microbial population development and increase nutrient absorption. Hence, the dietary inclusion of thymol into chicken diets stimulated lipase activity by 29% and trypsin activity by 18% in the digestive tract. Despite the fact that our feed efficiency parameters results revealed no significant variations in feed efficiency (TFI and FCR) because of the dietary supplementation of PLE on the broiler diets, but the lowest FI and FCR values were reported in the broiler-fed diets supplemented with higher PLE levels. In the same context, the numerically decreasing FCR values and mortality percentage highly correlated with the higher EPEI values in the broiler treatments supplemented with graded amounts of PLE extract. This finding was reported to be in agreement with Olaifa et al. (41) and Elbaz et al. (42), who stated that phytogenic feed additives could significantly improve all performance and feed efficiency parameters, namely, live body weight, weight gain, feed consumption, and feed conversion ratio of the broiler chickens. In addition, El-Ashram and Abdelhafez (43) reported that dietary inclusion of phytogenic feed additives such as thymol into the broiler diet significantly improved values of EPEF for the broiler compared with the untreated treatment. The antibacterial features of thymol and other flavonoids compounds found in PLE extract (14), as well as its capability to promote nutrient digestion (15), enhance intestinal structure, and increase epithelial absorption activity (12), may be responsible for the improvement in all feed efficiency indicators.

On the other hand, the blood biochemical components are usually correlated to the health conditions of birds (6). Furthermore, the alteration of biochemical parameters is a useful indicator of the nutritional, physiological, and pathological condition of the birds (44). In addition, it might be a valuable technique for determining the impact of feed additives on bird health and productivity (45). In the current study, all the biochemical blood markers were shown to be unaffected with PLE dietary administration, with the exception of triglyceride concentrations. The unchanged serum parameters were in line with the results of Al-Sagheer et al. (21) and Kovitvadhi et al. (46), who mentioned that dietary phytogenic supplementation had no significant effect on the biochemical parameters (total protein, globulin, and albumin) in growing rabbits. The absence of variations in these parameters suggests that dietary paulownia leaves extract supplementation has health advantages. Conversely, increasing the PLE level in the broiler diet significantly lowered the triglyceride levels in the blood. Recent studies have proven that thyme can decrease triglyceride and total cholesterol levels (47, 48). Yu et al. (49) reported that thymol possesses a lipid-reducing function by altering hepatic triglyceride secretion. As a consequence, our findings indicated that PLE dietary inclusion had no harmful effects on biochemical blood markers and could be applied safely.

Moreover, oxidative stress remarks such as GSH, SOD, MDA, and CAT are frequently used as indicators for assessing oxidative stress status (50). Regarding antioxidative biomarkers, supplementation of PLE improved the activity of antioxidant enzymes, namely, GSH, SOD, and CAT and decreased MDA levels in comparison with the unsupplemented treatment. These findings are consistent with several earlier studies which documented the antioxidant properties of *P. tomentosa* and its phenolic treatment constituents (15, 16, 35). Moreover, Alagawany et al. (12) showed that the dietary supplementation of phytogenic constituents significantly increased serum antioxidant enzyme activities and decreased MDA concentration. Herbal extract such as paulownia is a good source of many phytochemicals such as thymol and phytol and other bioactive components (28). These bioactive compounds found in *P. tomentosa* possess potent anti-inflammatory and antioxidant effects (4, 12, 16, 35). From these findings, it could be proposed that supplements with natural antioxidants could be practical in the future to enhance the health status of the broiler.

During evolution, ontogeny and immune responses, immunoglobulin (Ig) M is found to be the first isotype of antibodies that significantly stimulate (42). IgM not only acts as the first defensive line for the host against infectious pathogens, but it also plays a crucial role in inflammatory processes and innate immune responses (45). IgG antibodies are present in extracellular fluid and blood stream, where they might absorb pollutants, phagocytic viruses, and bacteria and stimulate the complement pathways (20). Our findings showed that supplementing birds' rations with PLE significantly stimulated immunoglobulin activity. In full agreement with our results, Yang et al. (17) and Wang et al. (20) reported that *paulownia tomentosa* flower (PTFP) as a new immunostimulant enhanced the humoral and cellular responses in chickens as well as promoted specific immunoglobulin (IgGs) antibodies responses in rats (16). The enhancement of immunoglobulin (IgM and IgG) in PLE supplemented treatments might be due to the antibacterial, antioxidant, and anti-inflammatory properties of bioactive components found in PLE (21). Thymol is the main component of PLE and it has been known for its positive immune effects such as an increase in lymphocyte proliferation rate, phagocytic rate as well as an increase in immunoglobulins such as IgA and IgM in the blood (51, 52).

Owing to our results, the expression of hepatic *IGF-I* and *GHR* mRNA significantly improved in birds fed supplemented diets with graded amounts of PLE. Similar results have been reported by Hosseini et al. (53), who concluded that using phytogenic feed additives (such as Thymolina) in the broiler chicken's diet causes improvements in the immune system by increasing the gene expression of hepatic *IGF-1*. Thus, the upregulation of hepatic *IGF-I* and *GHR* mRNA levels of birds fed PLE supplemented treatments might be due to the growth-promoting properties of the bioactive components found in PLE, specifically thymol and phytol (51). For the same reason, anti-inflammatory and immunomodulatory properties of bioactive components found in paulownia extract have the ability to upregulate mRNA expression of spleen pro-inflammatory interleukins (*IL-1*β and *IL-10*) (53). It is well-known that the expressions of interleukin *IL-1*β and *IL-6* are highly associated with the immune status of poultry and livestock (6). Consequently, thymol as the major compound found in paulownia leaves extracts significantly upregulated the expression of pro-inflammatory cytokines, namely, *IL-1*β*, IL-6, IL-10, IL-12*α, and *IL-18* (54).

## Conclusion

Dietary supplementation of PLE significantly improved FBW, WG, FCR, while remarkedly enhanced EPEI values. However, the treatment increases the activity of oxidative remarks (GSH, SOD, and CAT). Besides, our study revealed the dose-dependent effect of PLE on serum immunoglobulin (IgM and IgG) activity in broiler. Also, a dramatic linear increase was observed in mRNA expression of hepatic (*IGF-1* and *GHR*) and splenic (*IL-1*β and *IL-10*) of broiler chickens. The current research revealed that PLE at 0.5 g per kg diet is the most acceptable dosage for supplementation in broilers to optimize performance, improve oxidative remarks activity, and boost immunity without affecting productivity. However, larger cohort studies are needed to provide mechanistic insights into the role of PLE in alleviating oxidative stress, modulating intestinal microbial biodiversity, and mediating metabolic activities associated with gut function activity in the broiler chicks.

## Data Availability Statement

The original contributions presented in the study are included in the article/supplementary material, further inquiries can be directed to the corresponding author/s.

## Ethics Statement

The animal study was reviewed and approved by all *in-vivo* trials were followed the practical guidelines of the Local Experimental Animal Care Committee and approved by the Ethics Committee of Animal Use in Research Committee of Mansoura University (Code No: R/87).

## Author Contributions

SS, HE-E, NW, ME, YA, SA, MSo, and HZ carried out broiler maintenance and sample collection and contributed to conception and design of the study. MN, MA, YA, SA, MSo, and MSh organized the database and performed the statistical analysis. MN and SA drafted the manuscript and prepared it for publishing. SS, HE-E, MN, NW, HZ, MA, YA, SA, MSo, MSh, and ME read, agreed, and equally established the final published version of the manuscript. All authors contributed to the article and approved the submitted version.

## Funding

This study was supported by the Taif University Researchers Supporting Project (TURSP-2020-258), Taif University, Taif, Saudi Arabia.

## Conflict of Interest

The authors declare that the research was conducted in the absence of any commercial or financial relationships that could be construed as a potential conflict of interest.

## Publisher's Note

All claims expressed in this article are solely those of the authors and do not necessarily represent those of their affiliated organizations, or those of the publisher, the editors and the reviewers. Any product that may be evaluated in this article, or claim that may be made by its manufacturer, is not guaranteed or endorsed by the publisher.
